# Phosphorylation of p65(RelA) on Ser^547^ by ATM Represses NF-κB-Dependent Transcription of Specific Genes after Genotoxic Stress

**DOI:** 10.1371/journal.pone.0038246

**Published:** 2012-06-08

**Authors:** Hélène Sabatel, Emmanuel Di Valentin, Geoffrey Gloire, Franck Dequiedt, Jacques Piette, Yvette Habraken

**Affiliations:** 1 Laboratory of Virology and Immunology, GIGA-R, Signal Transduction Unit, University of Liège, Liège, Belgium; 2 Interface Entreprises-Université Liège Science Park, Angleur, Belgium; 3 Laboratory of Signalisation and Protein Interaction, GIGA-R, Signal Transduction Unit, University of Liège, Liège, Belgium; Johns Hopkins School of Medicine, United States of America

## Abstract

The NF-κB pathway is involved in immune and inflammation responses, proliferation, differentiation and cell death or survival. It is activated by many external stimuli including genotoxic stress. DNA double-strand breaks activate NF-κB in an ATM-dependent manner. In this manuscript, a direct interaction between p65(RelA) and the N-terminal extremity of ATM is reported. We also report that only one of the five potential ATM-(S/T)Q target sites present in p65, namely Ser^547^, is specifically phosphorylated by ATM *in vitro*. A comparative transcriptomic analysis performed in HEK-293 cells expressing either wild-type HA-p65 or a non-phosphorylatable mutant HA-p65_S547A_ identified several differentially transcribed genes after an etoposide treatment (e.g. IL8, A20, SELE). The transcription of these genes is increased in cells expressing the mutant. Substitution of Ser^547^ to alanine does not affect p65 binding abilities on the κB site of the IL8 promoter but reduces p65 interaction with HDAC1. Cells expressing p65_S547A_ have a higher level of histone H3 acetylated on Lys^9^ at the IL8 promoter, which is in agreement with the higher gene induction observed. These results indicate that ATM regulates a sub-set of NF-κB dependent genes after a genotoxic stress by direct phosphorylation of p65.

## Introduction

Genomic integrity is continuously challenged by endogenous and external threats such as genotoxic chemicals or ionizing radiation (IR). DNA damage, and in particular DNA double-strand breaks (DSB), generated by these agents, are extremely deleterious. Indeed they can lead to genetic variation, aging, cell death or carcinogenesis. Cells activate a complex signaling network, called the DNA Damage Response (DDR), to respond to those injuries and avoid such consequences [1], [2]. Sensor proteins detect DNA lesions and transmit the signal to transducer proteins which, in turn, convey the signal to numerous downstream effectors involved in specific pathways. Upon DSB formation, the main transducer protein of the DDR is Ataxia telangiectasia mutated (ATM), a large mostly nuclear kinase. Indeed, upon detection of DSB, ATM is rapidly activated and can next phosphorylate a vast amount of substrates implicated in DSB repair, check-point activation, apoptosis and transcription factors regulation, including p53 and the nuclear factor kappa B (NF-κB) [3], [4], [5].

NF-κB is an important regulator of diverse cellular processes, including immune response, inflammation, cell survival, proliferation, differentiation, adhesion and apoptosis [6], [7]. Aberrant activation of NF-κB pathway has been implicated in many pathogenesis. In particular, NF-κB constitutive activation contributes to the growth and malignancy of cancer cells and also affects the tumor response to many types of chemotherapy and ionizing radiation. It often favors survival and thereof the apparition of detrimental resistance phenomenon [8], [9].

NF-κB family includes five members, p65(RelA), RelB, c-Rel, p105/p50 and p100/p52, which assemble in homodimers or heterodimers to form active transcription factors that regulate a wide array of target genes. The NF-κB p50–p65 complex represents the most abundant member of the NF-κB family [10]. In this dimer, p65(RelA) possesses a transactivating function while p50 does not. In resting cells, NF-κB complexes are bound to inhibitor of NF-κB (IκB) proteins such as IκBα, and remain inactive in the cytoplasm. Distinct signaling cascades, induced by various stimuli such as tumor necrosis factor α (TNFα), lipopolysaccharide (LPS) or genotoxic agents, activate NF-κB pathway via the IκB kinase (IKK) complex activation. This complex is composed of two catalytic subunits (IKKα and IKKβ) and one regulatory subunit named IKKγ or NEMO for NF-κB essential modulator [11]. In response to DSB, ATM kinase is required for IKK activation at two levels. On the one hand, ATM phosphorylates NEMO in the nucleus [12]. This phosphorylation requires the previous PIASy-mediated sumoylation of NEMO and leads to subsequent NEMO mono-ubiquitination and nuclear export. These sequential modifications of NEMO are essential for IKK complex activation [13]. On the other hand, following DSB, ATM is partially exported to the cytoplasm where it allows TGF-beta activated kinase 1 (TAK1) activation and TNF receptor-associated factor 6 (TRAF6) poly-ubiquitination, two additional steps also required for full IKK complex activation [14], [15]. Once activated, the IKK complex phosphorylates IκBα, leading to its recognition by the SCF^βTrCP^ and resulting in its poly-ubiquitination and subsequent degradation by the 26S proteasome. NF-κB released from IκBα inhibitor translocates to the nucleus, binds to DNA and regulates genes expression [11].

NF-κB activity is controlled by highly regulated mechanisms. Indeed, post-translational modifications of NF-κB subunits also modulate gene transcription. p65 is phosphorylated on different residues (e.g. Ser^276^, Ser^311^, Ser^468^, Thr^505^, Ser^529^, Ser^536^) by multiple kinases (e.g. PKAc, MSK1, GSK3β, Chk1, CKII, RSK1, IKKε, IKKα, IKKβ) in response to diverse stimuli, and these phosphorylations were shown to have an effect on NF-κB activity [16], [17]. Indeed they can affect (i) NF-κB nuclear translocation, (ii) DNA binding or (iii) gene transcription. p65 phosphorylations can modulate positively as well as negatively gene induction, by increasing or decreasing co-activator and co-repressor recruitment. Moreover, p65 can be reversibly acetylated by histone acetyltransferase (HAT) (e.g. CBP/p300, PCAF) on multiple residues (Lys^122^, Lys^123^, Lys^218^, Lys^221^, Lys^310^, Lys^314^ and Lys^315^). p65 acetylations also play different roles in NF-κB activation [16], [17]. Through the study of p65 post-translational modifications, the molecular mechanisms underlying the regulation of specific target genes are better understood and reveal a growing complexity.

This study shows that ATM kinase directly phosphorylates p65 on Ser^547^ in response to DSB. This interaction between ATM and p65 highlights a third level of NF-κB regulation by ATM kinase. In addition, it also demonstrates that this phosphorylation decreases the expression of a specific set of NF-κB target genes implicated in inflammation, by a mechanism involving histone deacetylase (HDAC) recruitment.

## Results

### Interaction between ATM and p65 and Phosphorylation of p65 by ATM

ATM is the main mediator of the DSB-induced DDR, therefore the identification of new binding proteins would help in the understanding of the signaling pathways induced by DNA breaks. In order to discover new interacting proteins, a yeast two-hybrid screen was performed. ATM (1–150) (HEAT repeats 1–2) was used as bait and a HeLa cDNA library as prey. The N-terminal part of ATM was chosen because it is known to interact with other proteins and substrates [18]. Twelve different proteins, including ATM itself and p65, were identified as potential ATM-binding proteins. In an X-Gal filter test, an intense blue coloration rapidly developed in the colony expressing p65 while this coloration was weaker and slower to develop in the colony expressing ATM. These results suggested a strong interaction between ATM and p65. The presence of ATM, known to form homodimer, among the prey validated the screen. Given the important roles of ATM kinase in DSB-induced NF-κB pathway, p65(RelA) seemed to be of great interest for further studies.

**Figure 1:**
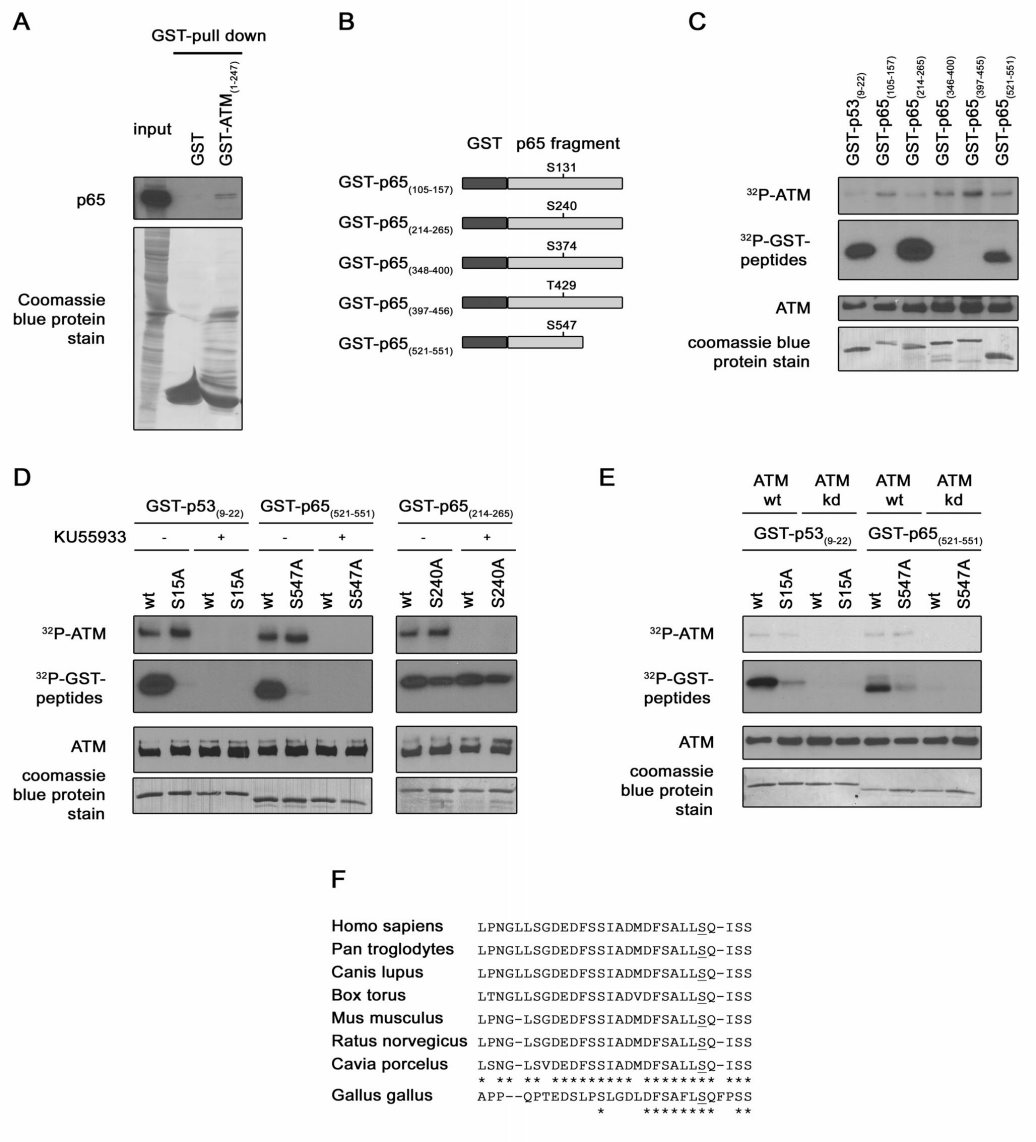
p65 interaction with ATM and p65 Ser^547^ phosphorylation by ATM. (**A**) Confirmation of a direct interaction between ATM and p65. Bacterially expressed GST or GST-ATM (1–247) fusion protein were purified on glutathione agarose beads and used to pull-down p65 from HEK-293 over-expressing HA-p65 cell lysate. Pulled down p65 was detected by immunoblotting with a p65 antibody (upper panel) and GST proteins were stained with coomassie bleu on the PVDF membrane. (**B**) Identification of the ATM target residue. Schematic representation of the different GST-p65 substrates used in the kinase assay. (**C**) *In vitro* kinase assay. Immunoprecipitated ATM from HEK-293 cells was incubated with GST-p53 and different GST-p65 proteins in presence of [γ−^32^P]ATP. The radiolabelled bands (upper panels) represent auto-phosphorylated ATM, phosphorylated GST-p53 or GST-p65. Levels of ATM and of substrate present in each reaction were determined by western blotting and by coomassie blue staining respectively (lower panels). (**D**) As in (C) *In vitro* kinase assay but with wt or mutated GST-p53 and GST-p65 proteins as substrates. ATM inhibitor KU55933 was added in some reaction samples as indicated. The same detection methodology than in (**C**) was used. (**E**) As in (D) *in vitro* kinase assay, but with purified recombinant ATM wt or kd instead of immunoprecipitated ATM and KU55933 utilization. (**F**) Conservation of Ser^547^ among different species. Alignment of p65 C-terminal sequence from different mammalian and bird species.

In a first time the interaction between ATM N-terminal extremity and p65 was investigated by GST pull-down assay, using purified GST-ATM_(1–247)_ fusion protein. Cellular extracts from cells over-expressing HA-p65 were mixed with GST-ATM or with GST alone as negative control. These results showed an interaction between p65 and GST-ATM_(1–247),_ but not between p65 and GST, confirming the existence of a stable interaction between p65 and ATM (Fig. 1A).

ATM is a kinase with multiple substrates [19]. In order to investigate whether p65 could be one of them, an *in silico* analysis of p65 primary structure was performed. This analysis revealed five (S/T)Q motives (Ser^131^, Ser^240^, Ser^374^, Thr^429^, Ser^547^). This motif is the minimal established consensus sequence targeted by ATM kinase [20]. The phosphorylation of these residues was investigated by an *in vitro* kinase assay using different purified GST-p65 fusion proteins as substrates. The different GST-p65 fusion proteins contain between 30 to 50 amino acids of p65 surrounding each candidate phosphorylation site (Fig. 1B). These GST-p65 proteins were incubated with immunoprecipitated active ATM and [γ−^32^P]ATP. A phosphorylation of the substrates containing the Ser^240^ and the Ser^547^ was observed (Fig. 1C). Two additional assays were conducted to confirm the specificity of the detected radioactive signal. The kinase assay was repeated (i) with mutated GST-p65 fusions proteins, in which the putative targeted serine was replaced by an alanine, or (ii) in presence of a specific ATM kinase inhibitor (KU55933). These experiments showed a specific phosphorylation of Ser^547^ by ATM kinase but not of Ser^240^. Indeed, the phosphorylation of the substrate disappeared when mutated GST-p65_(531–551)_ was used or when the inhibitor KU55933 was added in the reaction sample (Fig. 1D left panel), whereas, for the substrate containing the Ser^240^, it did not (Fig. 1D right panel). Ser^15^ of p53 being a well known target of ATM [21], GST-p53_(9–22)_ wt or mutated were taken as controls. The lack of ATM auto-phosphorylation in presence of the inhibitor further validates this test (Fig. 1D top panels). Moreover, to confirm the phosphorylation of the Ser^547^ of p65 by ATM kinase by an independent assay, an experiment was performed using purified recombinant ATM wt or kd instead of the immunoprecipitated kinase. This additional kinase assay also showed a specific phosphorylation of the Ser^547^ of p65, confirming completely the specificity of the signal observed to ATM kinase (Fig. 1E). From these experiments, we can conclude that the p65 subunit of NF-κB interacts with ATM to be phosphorylated on its Ser^547^. This residue is located in the transactivation domain 1 (TAD1) of p65 at the C-terminal extremity. It is evolutionary conserved and found in mammalian and in bird species too (Fig. 1E) [22].

### Role of p65 Ser^547^ Phosphorylation on Global Transcriptional Activity

**Figure 2:**
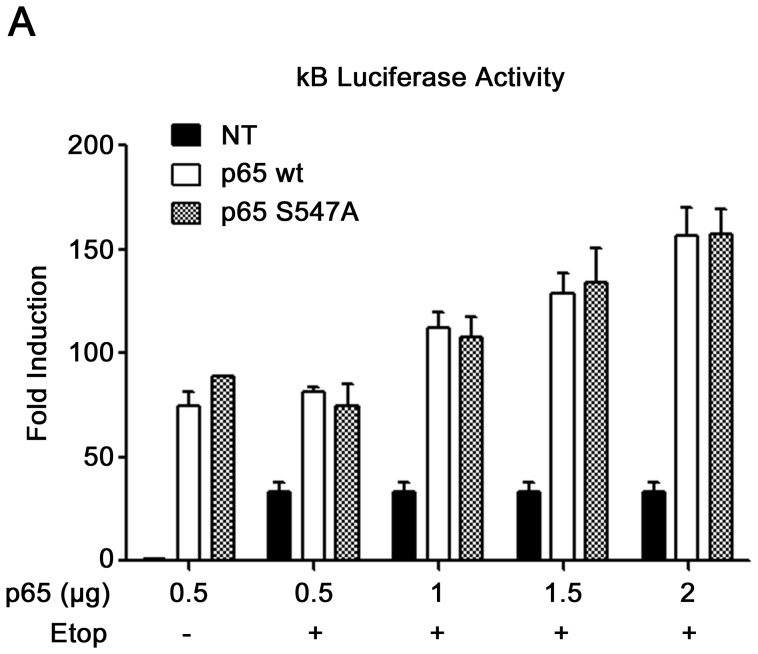
No impact of p65 Ser^547^ phosphorylation on global NF-κB transcriptional activity. (**A**) HEK-293 cells were transfected with κB Luciferase plasmid and with increasing amount of either wt or S547A mutated p65. Twenty-four hours later the cells were treated for 8 h with etoposide or left untreated. NT, non transfected.

To investigate the effects of p65 Ser^547^phosphorylation on transcriptional NF-κB activity, a phospho-null (S547A) mutation of this site was generated. HEK-293 cells, transfected either with p65_wt_ or p65_S547A_ expression plasmids and luciferase reporter plasmids, were treated with etoposide to induce the DDR cascade. The results indicate an expected increased luciferase activity of the non-transfected cells treated with etoposide, but they did not reveal any difference in luciferase activity between cells over-expressing wt or S547A mutated p65 (Fig. 2A). Over-expressed p65 wt or mutated are both able to induce NF-κB activity independently of etoposide treatment. Nevertheless, these results indicate (i) that the mutant does not exercise a dominant negative effect and (ii) that the phosphorylation of this residue does not have a global impact on the transcriptional potential of p65. However Ser^547^ phosphorylation could have a gene specific effect. Indeed, several other p65 post-translational modifications are known to have gene specific effects [16], [23], [24].

### HEK-293 Cell Lines Stably Expressing Either p65_wt_ or p65_S547A_

**Figure 3:**
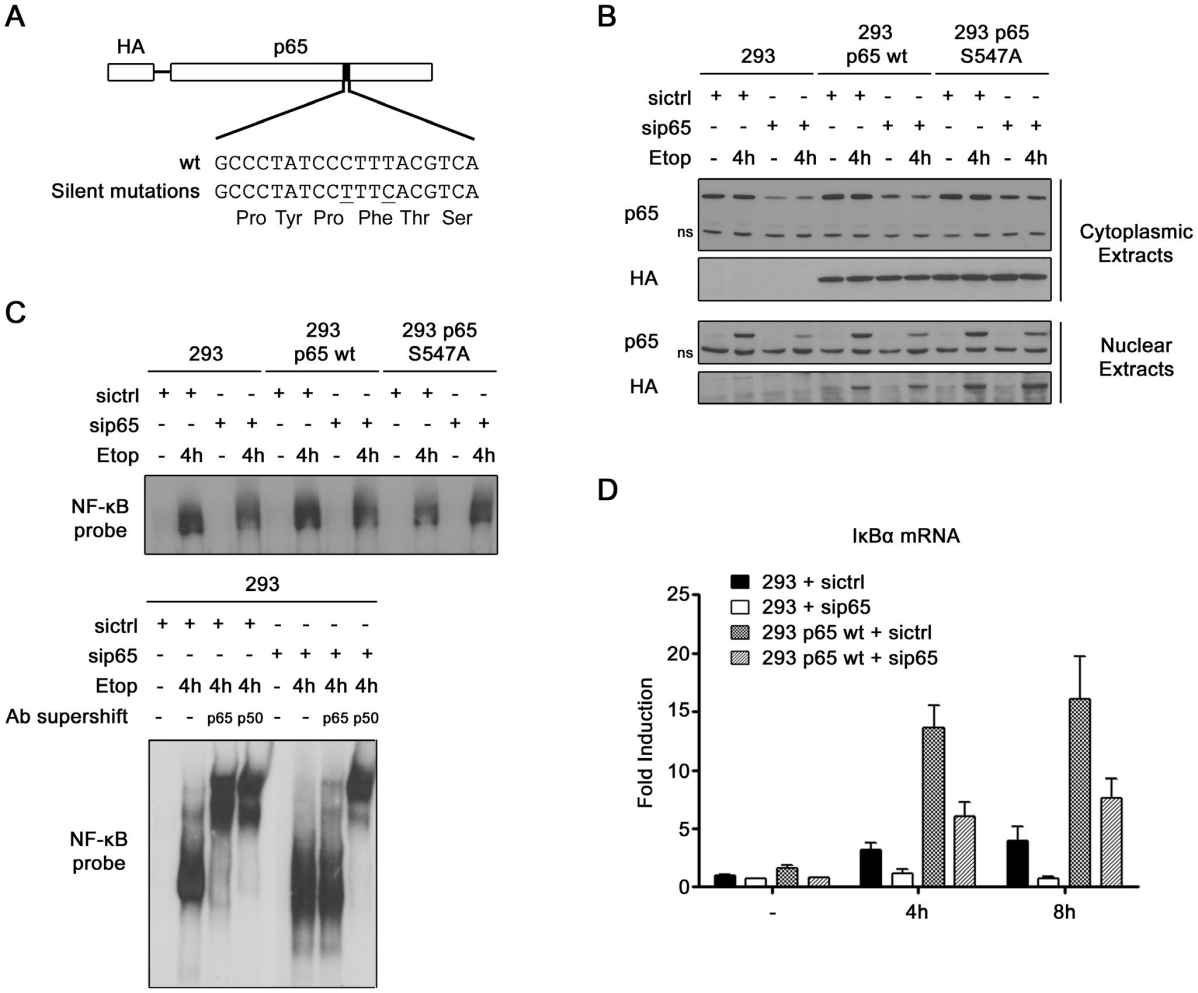
Creation of HEK-293 cell lines expressing either wt or S547A mutated p65. (**A**) Schematic representation of the 2 silent mutations inserted in the sequence of p65 within the sequence targeted by p65 siRNA, conferring to exogenous HA-p65 the resistance to the siRNA. (**B**) Western blot analysis of cytoplasmic and nuclear p65 protein (p65 antibody) and of exogenous p65 (HA antibody) in non-treated or etoposide treated HEK-293 and HEK-293 stably expressing sip65 resistant HA-p65. All cell lines were either transfected with control or p65 siRNA. (**C–D**) Comparison of NF-κB activation after etoposide treatment, by (C) EMSA and by (D) qRT PCR of IκBα mRNA, between HEK-293 and HEK-293 stably expressing sip65 resistant HA-p65. All cell lines were transfected either with control or p65 siRNA. (**C**) NF-κB DNA binding was analyzed using a probe corresponding to the HIV-1 long terminal repeat κB site (top panel). The presence of p65 and p50 protein in the binding complex observed in HEK-293 was analyzed by supershift with p65 and p50 antibodies (bottom panel). ns, non specific band.

To study the physiological role of Ser^547^ phosphorylation, a cellular model which activates NF-κB in response to DSB and which expresses either wt or S547A mutated p65 was created. HEK-293 cells were used and a strategy was adopted to down-regulate endogenous p65 and to re-introduce a HA-tagged wt or S547A p65 plasmid at an expression level similar to the endogenous one. Therefore, two silent mutations were inserted in the p65 sequence targeted by a p65 siRNA (Fig. 3A). HEK-293 cells were stably transduced by lentiviral infection with this siRNA resistant HA-p65, and endogenous p65 was next down-regulated with siRNA. Western blot experiments showed that endogenous p65 was down-regulated by the p65 siRNA whereas the wt and S547A HA-p65 were not (Fig. 3B). An inducible translocation of HA-p65 was also observed in response to etoposide, demonstrating the functionality of the exogenous p65. To further check the functionality of this cellular model, EMSA (Fig. 3C) and qRT PCR (Fig. 3D) were performed. By EMSA, NF-κB binding in response to DNA damage was observed, as expected (Fig. 3C top panel). The residual binding observed in HEK-293 cells depleted in endogenous p65 corresponds to p50/p50 binding, as shown by the supershift experiment (Fig. 3C bottom panel). Quantitative analysis of IκBα mRNA level showed the inducible expression of NF-κB target genes in response to etoposide in the model, demonstrating once again its functionality (Fig. 3D). Moreover, the qRT PCR experiment showed that, even if endogenous p65 was not completely knocked out by the siRNA, the residual amount of endogenous p65 protein is no longer sufficient to induce gene expression. In regard of these data, we obtained a functional cellular model, expressing exclusively wt or S547A mutated p65, which activates NF-κB in response to DSB.

### Regulation of a Set of Specific NF-κB Target Genes by p65 Ser^547^ Phosphorylation

**Figure 4:**
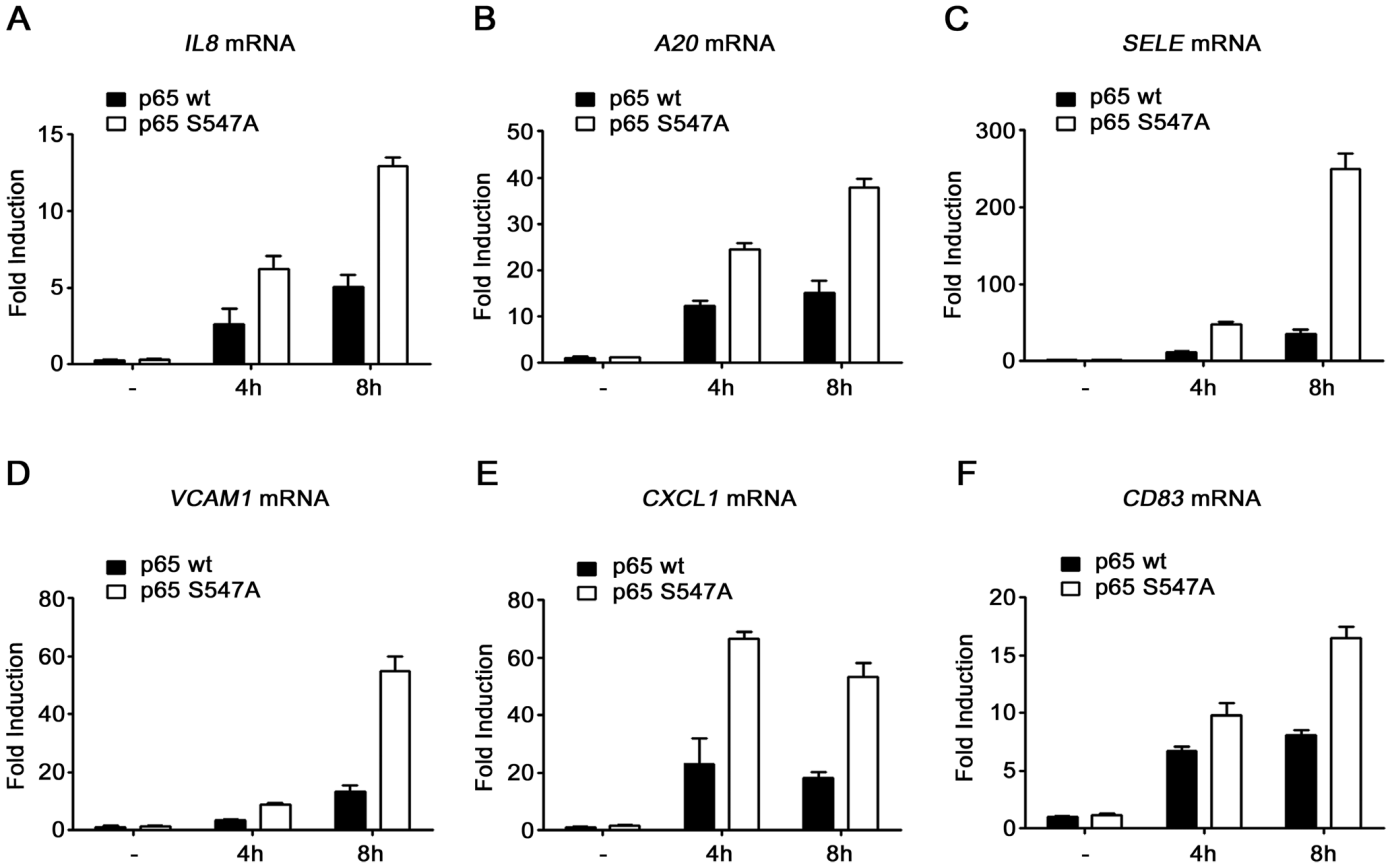
Higher expression of specific genes in cells expressing S547A mutated p65. qRT PCR analysis of *IL8* (**A**), *A20* (**B**), *Sele* (**C**), *VCAM1* (**D**), *CXCL1* (**E**) and *CD83* (**F**) mRNA level after 4 and 8 hours of etoposide treatment in HEK-293 cells expressing either p65_wt_ or p65_S547A_.

**Figure 5:**
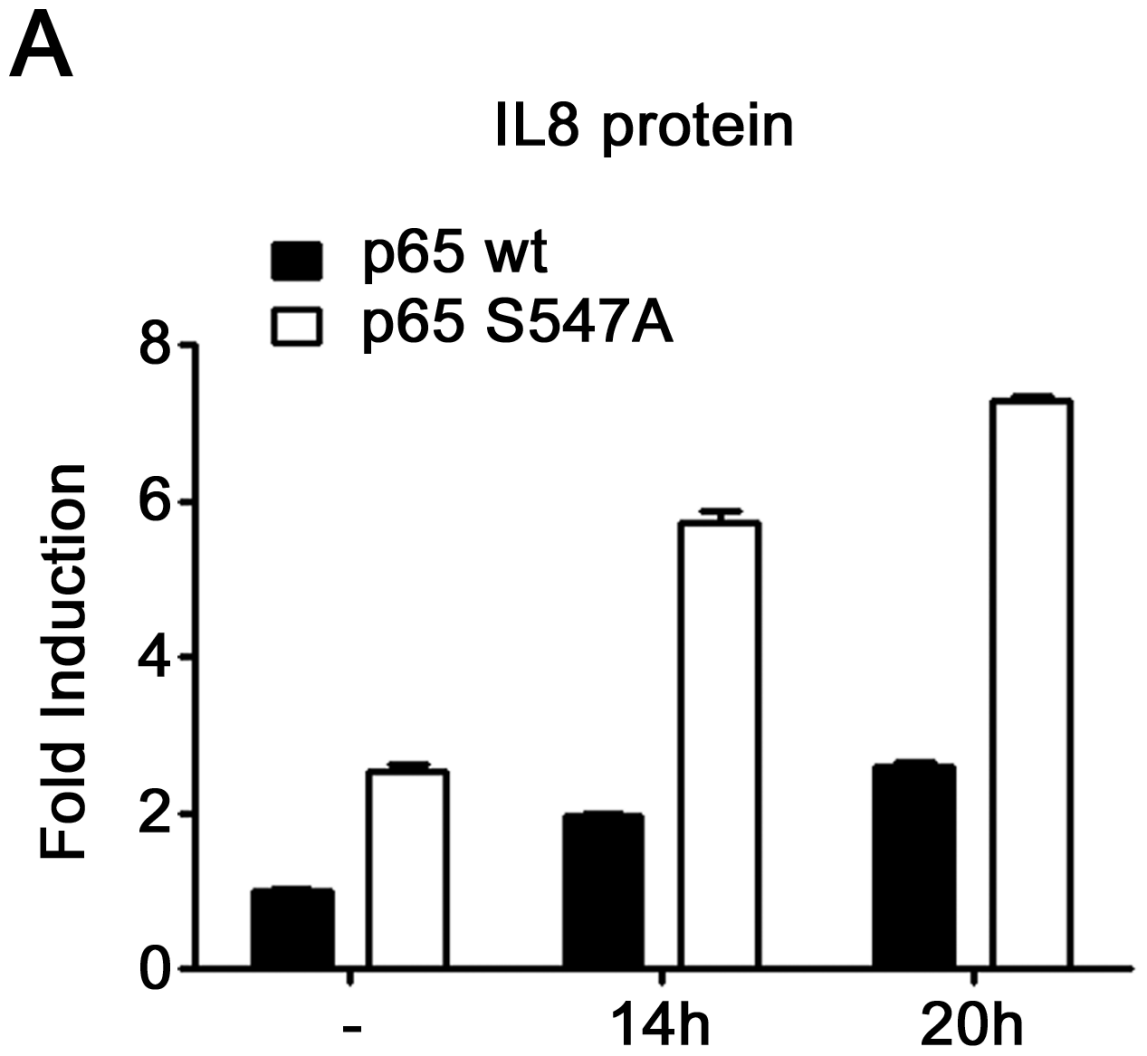
Higher IL8 protein level in cells expressing S547A mutated p65. ELISA analysis of IL8 protein level after the indicated times of etoposide treatment in HEK-293 cells expressing either p65_wt_ or p65_S547A_.

**Figure 6:**
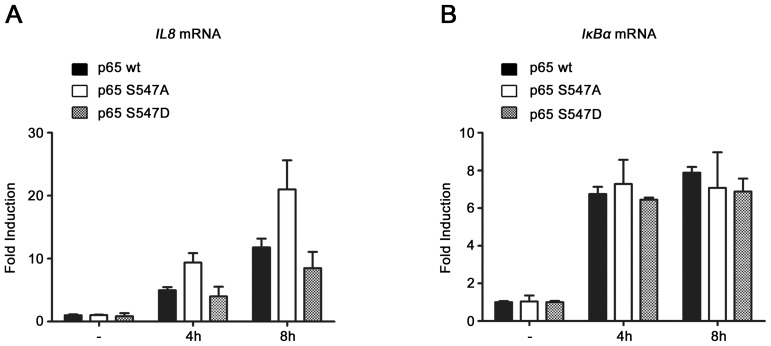
Similar etoposide-induced gene expression profile in cells expressing wt and S547D mutated p65. qRT PCR analysis of *IL8* (A), and *IκBα* (B) mRNA level after 4 and 8 hours of etoposide treatment in HEK-293 cells expressing either p65_wt_, p65_S547A_, or p65_S547D_.

A transcriptomic analysis by microarray Illumina was performed, in this cellular model after 4 and 8 hours of etoposide treatment, in order to identify the genes specifically regulated by the Ser^547^ phosphorylation of p65 in response to DSB. The microarray revealed a group of around fifteen genes differentially regulated between cells expressing wt and S547A mutated p65 (Tab 1). The expression level of these genes was always higher in cells expressing S547A mutated p65 compared to wt p65, indicating a possible repressor role for the phosphorylation of the Ser^547^. Most of these genes were already described as NF-κB-dependent genes and often implicated in inflammation. The different mRNA expression level between both cell types was confirmed by qRT PCR for some of the genes (Fig. 4A–F). The results of the microarray were further validated at the protein expression level by ELISA for interleukin 8 (IL8) chosen as model for further investigations (Fig. 5A). To prove that the higher gene induction observed in cells expressing p65_S547A_ is effectively due to the lack of phosphorylation of this residue, a HEK-293 cell line expressing exclusively a phospho-mimicking (S547D) mutant of p65 was obtained. Etoposide-induced IL8 and IκBα gene expression was next analyzed in these cells by qRT PCR (Fig. 6A and 6B). Interestingly, no difference of IL8 gene induction was observed between cells expressing the wt and the S547D mutant form of p65, whereas the higher gene expression was again observed in cells expressing p65_S547A_. As expected, IκBα, which was not among the set of genes regulated by the phosphorylation of the Ser^547^ of p65, showed an identical induction in the three different cell lines. In conclusion, p65 phosphorylation on Ser^547^ in response to DSB leads to a decreased induced expression of a specific set of genes mainly involved in inflammation.

**Table 1:** Genes regulated by p65 Ser^547^ phosphorylation.

	p65 wt		p65 S547A	
Gene Symbol	Etop 4 h (FI)	Etop 8 h (FI)	Etop 4 h (FI)	Etop 8 h (FI)
SELE	2,30	4,82	5,51	18,73
A20	5,89	7,07	11,66	14,47
IL8	3,19	5,31	6,22	11,51
PI3	2,43	4,57	4,65	9,60
CXCL2	1,49	1,17	2,77	1,97
CXCL1	3,38	2,47	6,02	4,05
CD83	3,25	3,94	5,19	6,45
CCL20	11,55	39,21	18,37	52,53
GADD45B	2,68	2,12	4,18	2,33
TNF	4,27	5,47	6,20	7,03
VCAM1	1,06	1,84	1,42	3,83
BIRC3	1,38	2,33	1,83	3,47
C1QTNF1	1,25	1,57	1,36	2,50
SGPP2	1,04	2,81	1,12	4,38

List of genes differentially expressed in HEK293 cells expressing exclusively wt or S547A mutated p65, and their fold induction (FI) after treatment with etoposide. Gene symbol in the first column, fold induction after etoposide treatment for the indicated periods (compared to non-treated cells) and in the indicated cell line, in the following columns. Data obtained from the microarray experiment analysis.

### Mechanism of Gene Expression Down-regulation by p65 Ser^547^ Phosphorylation

**Figure 7:**
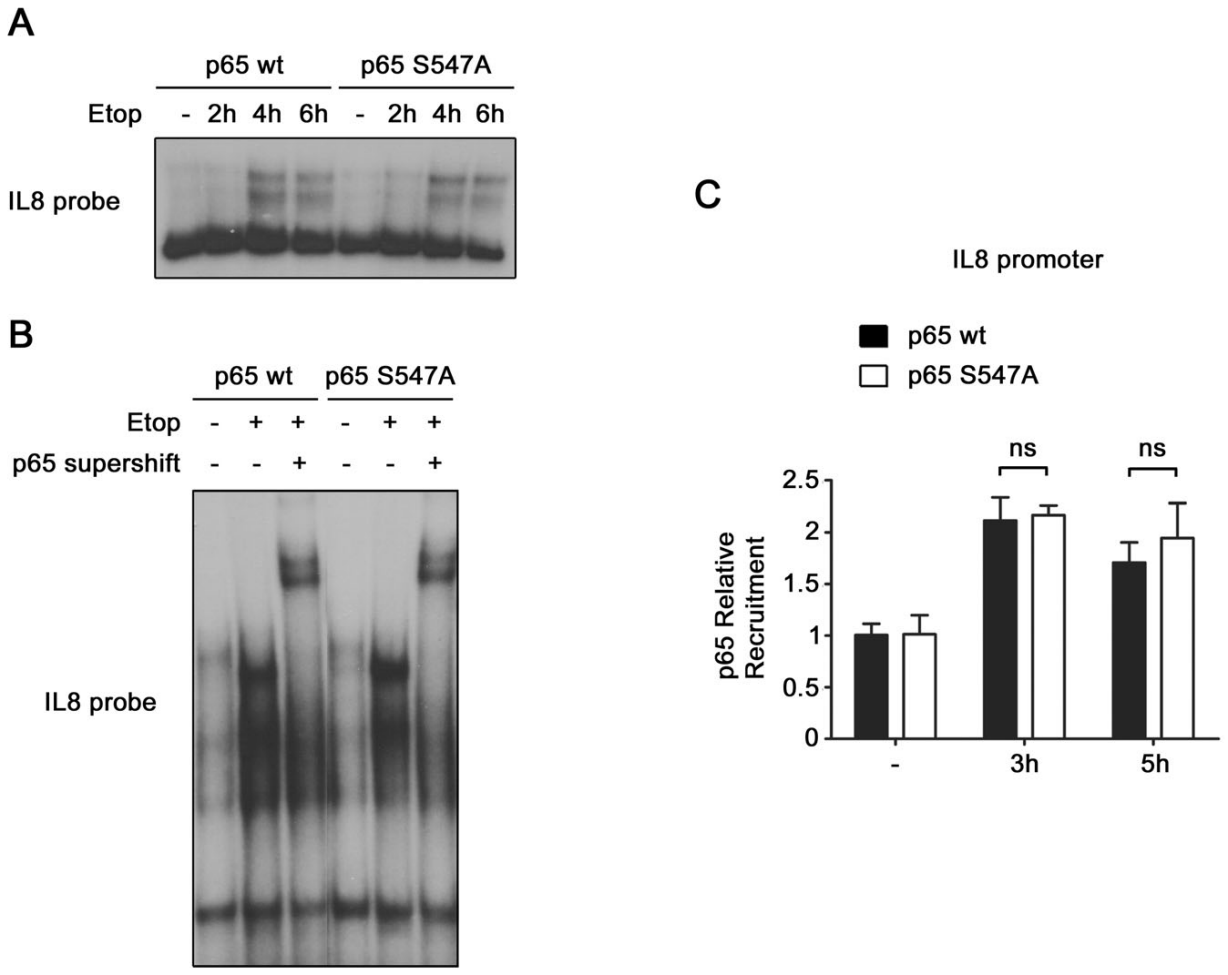
Identical binding of wt and S547A mutated p65 to IL8 κB site. (**A**) NF-κB binding to IL8 promoter, in HEK-293 cells expressing either p65_wt_ or p65_S547A_, non-treated or treated with etoposide, was analyzed by EMSA with a probe corresponding to the κB site of IL8 promoter. (**B**) The presence of p65 protein in the binding complex observed in (**A**) was analyzed by supershift with p65 antibody. (**C**) Recruitment of p65 on IL8 promoter was measured by p65 ChIP assay in HEK-293 cells expressing either p65_wt_ or p65_S547A_, and treated with etoposide for the indicated periods. ns, non significantly different.

Next, we investigated how p65 Ser^547^ phosphorylation could affect the transcription of this set of genes. To perform this study we focused on IL8 gene. The etoposide-induced binding of wt and S547A mutated p65 to IL8 promoter were compared by EMSA (Fig. 7A), and by p65 ChIP assay (Fig. 7C). No difference was observed, ruling out the hypothesis of a lower gene induction being the result of a lower recruitment of phosphorylated p65 onto the promoter. For the EMSA experiment, a supershift by a p65 antibody was performed. It showed that the signal observed was due to a p65 containing complex, likely p50/p65 (Fig. 7B).

**Figure 8:**
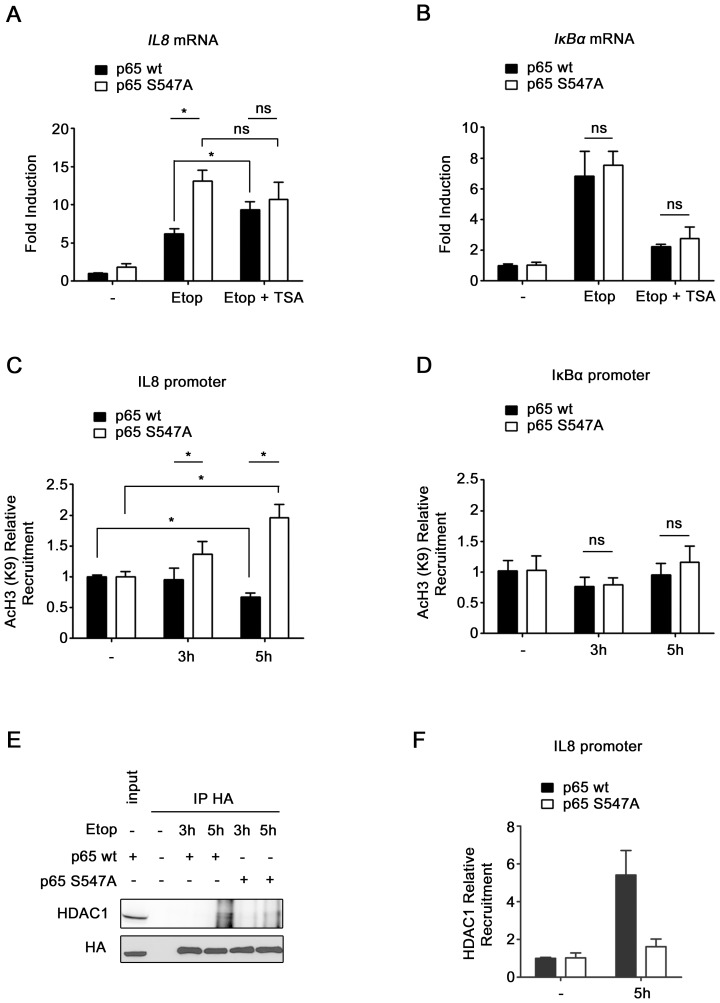
Ser^547^ phosphorylation–dependent IL8 gene regulation mechanism involve HDAC. (**A–B**) HEK-293 expressing either p65_wt_ or p65_S547A_ were non-treated or treated 4 hours with etoposide in absence or presence of TSA, and mRNA levels of IL8 (**A**) and IκBα (**B**) were analyzed by qRT PCR. (**C–D**) Recruitments of Lys^9^ acetylated histone 3 on IL8 (**C**) and IκBα (**D**) promoters were measured by ChIP assay in HEK-293 cells expressing either p65_wt_ or p65_S547A_, and treated with etoposide for the indicated periods. (**E**) HA-tagged p65_wt_ or p65_S547A_ were immunoprecipitated from lysates of HEK-293 cell transfected with expression plasmid for these two proteins or non transfected as control. Co-immunoprecipitated HDAC1 protein was detected by western blotting (upper panel). Level of immunoprecipitated HA-p65 in the different samples was determined by western blotting (lower panel). A portion of whole cell extract was added on the gel as input. (**F**) Recruitment of HDAC1 on IL8 promoter was measured by ChIP assay in HEK-293 cells expressing either p65_wt_ or p65_S547A_, and treated 5 hours with etoposide or left untreated. *, significantly different (P<0.05); ns, non significantly different.

A second possible explanation for the lower gene expression in cells containing wt p65 could be the recruitment of a co-repressor. HDAC are well-characterized gene expression repressors, known to be differentially recruited following specific p65 post-translational modifications. For this reason, HDAC recruitment following p65 phosphorylation was investigated. First, we observed the effect of trichostatin A (TSA) treatment, an HDAC inhibitor, on IL8 expression after etoposide treatment, in cells expressing wt or S547A mutated p65. Interestingly, TSA treatment increased etoposide-induced IL8 expression in cells containing wt p65 whereas it did not affect IL8 expression in cells containing S547A mutated p65 (Fig. 8A). This meant that HDAC probably affect IL8 expression only when p65 is phosphorylated. To confirm the specificity of the sensitivity to TSA for phosphorylated p65, we performed the same experiment with IκBα, a gene not regulated by p65 Ser^547^ phosphorylation. Surprisingly, it was observed that TSA also affected IκBα gene expression. However, the lower IκBα gene expression was observed both in cells containing p65_wt_ as well as p65_S547A_ (Fig. 8B). This indicated that IκBα gene expression following etoposide treatment was sensitive to TSA but, in the contrary of IL8 gene, in a p65 Ser^547^ phosphorylation-independent manner.

To confirm HDAC recruitment following p65 phosphorylation, we looked for the presence of acetylated histone on IL8 promoter in cells expressing wt and S547A mutated p65 by ChIP assay. ChIP experiment showed an increasing amount of acetylated histone 3 (Lys9) onto IL8 promoter following etoposide treatment in cells containing S547A mutated p65, and a decreasing amount of acetylated histone 3 in cells expressing wt p65 in the same conditions (Fig. 8C). The same experiment was performed with IκBα promoter on which no difference of histone 3 acetylation between cells expressing wt and mutated p65 was observed (Fig. 8D). This test showed almost no change of histone H3 acetylation following etoposide treatment on IκBα promoter. All of these results showed evidence for HDAC recruitment by phosphorylated p65 onto promoters of the genes specifically regulated by Ser^547^ phosphorylation. Next, co-immunoprecipitations between wt p65 or mutated p65 and HDAC were performed to compare potential differences in HDAC interaction. It can be seen on Fig. 8E that wt p65, but not mutated p65, interacts with HDAC1 after 5 hours of etoposide treatment. To further confirm the recruitment of HDAC1 on IL8 promoter by phosphorylated p65, HDAC1 ChIP assay was performed. This experiment showed a higher recruitment of HDAC1 after 5 hours of etoposide treatment in cells expressing wt p65 compared to cells expressing S547A mutated p65, corroborating the result of the co-immunoprecipitation (Fig. 8F). These data indicate that the phosphorylation of p65 on this residue following etoposide treatment leads to interaction with HDAC1.

All together these results demonstrate that, on specific promoters and in particular on IL8 promoter, phosphorylation of p65 by DSB-activated ATM leads to interaction with HDAC1 which deacetylates histones, leading to a decreased gene transcription.

## Discussion

The primordial role of ATM kinase in NF-κB activation by DSB was demonstrated some years ago [25], [26], but the different molecular mechanisms by which it controls this signaling cascade are continuously uncovered. In 2006, ATM was shown to phosphorylate NEMO in the nucleus [12]. More recently, it was demonstrated that ATM is also required in the cytoplasm for TAK1 activation and TRAF6 poly-ubiquitination [14], [15]. Our data show a third level of NF-κB regulation by ATM via the direct interaction with the NF-κB subunit p65 and its phosphorylation on the Ser^547^. The exact domain of p65 interacting with ATM was not investigated. Nevertheless, a direct interaction between the TAD1 domain of p65 and ATR or DNA-PK, two kinases belonging to the phosphoinositide-3-kinase like kinase (PIKK) family like ATM, has been reported [27]. As the targeted serine is localized in this domain, ATM probably also interacts with the TA1 domain of p65.

The kinase assays showed an incorporation of radioactivity in the GST-p65_(521–551)_ containing the Ser^547^, but also in the GSTp65_(214–265)._ This last signal was not ATM specific as it was still observed in presence of the ATM inhibitor and when the putative targeted serine (Ser^240^) is mutated. It indicates the presence of another kinase in the immunoprecipitate.

By phosphorylating p65, ATM acts downstream of IKK activation. Indeed, the Ser^547^ phosphorylation does not affect IκBα phosphorylation or p65 translocation (data not shown). Nevertheless the exact cellular compartment where Ser^547^ phosphorylation takes place is still unknown. Because this phosphorylation does not affect p65 translocation, we can hypothesize that it occurs in the nucleus. As Ser^547^ phosphorylation affects the transcription of only few specific genes, we can even assume that ATM could be recruited onto these specific promoters where it could phosphorylate p65. Further experiments are needed to explore this possibility.

For the study of the physiological impact of Ser^547^ phosphorylation following DNA damage, we could not use rescued MEF p65−/− with p65_wt_ or p65_S547A_, which would have been a quite simple model to use. Strikingly, p65 rescued MEFs, as well as independent MEFs p65+/+, do not activate NF-κB in response to DNA damage, even if TNF-induced NF-κB occurs normally in these cells (data not shown and discussed in [28]). That is why we choose HEK-293 cells in which NF-κB activation is observed in response to DNA damage. Nevertheless, over-expression of p65 induces NF-κB activation to a level too high to observe any further gene regulation following genotoxic stress (Fig. 2A and data not shown). Therefore, HEK-293 cells were transduced with a low amount of wt or S547A mutant p65 and endogenous p65 was down-regulated by siRNA. This adopted strategy provided an adequate cellular model for the study of p65 phosphorylation following genotoxic stress.

Phosphorylation of NEMO by ATM, and ATM-dependent TAK1 activation or TRAF6 polyubiquitination, are steps absolutely required for DSB-induced NF-κB activation [14], [15]. By contrast, Ser^547^ phosphorylation of p65 by ATM is a mechanism enabling a finely tuned NF-κB regulation. Indeed, this phosphorylation does not affect global NF-κB transcriptional potential, but regulates the expression of a set of specific genes. It is now largely accepted that the physiological role of NF-κB activation is quite complex and depends on the nature of the stimulus or on the cell type [17], [29]. Post-translational modifications, such as Ser^547^ phosphorylation, which are highly promoter-specific, could explain the specificity and selectivity required to produce this context-dependent NF-κB response. The microarray experiment identified a set of genes regulated by Ser^547^ phosphorylation mainly implicated in inflammation. Given the controversial but critical role of inflammation in tumor growth [30], [31], it would be interesting to analyze the physiological implications of Ser^547^ phosphorylation, on inflammation induction, following DNA damage.

Post-translational modifications of p65 can modulate NF-κB activity by different ways, and these mechanisms are context-dependent. For example, the phosphorylation of Ser^536^ by RSK1 was shown to facilitate NF-κB nuclear import by decreasing p65 affinity for IκBα [32], whereas the phosphorylation of the same residue by IKKα in response to LPS is required for the removal of p65 from NF-κB-dependent promoter [33]. p65 phosphorylations modulate also co-activator or co-repressor recruitment. Indeed, phosphorylation of Ser^276^ of p65 facilitates CBP/p300 recruitment to the promoters of NF-κB target genes [34]. On the opposite, Thr^505^ phosphorylation leads to HDAC recruitment and subsequent gene repression [23], whereas phosphorylation of Thr^435^ decreases p65 interaction with HDAC1, enhancing gene expression [24].

Here we observe that Ser^547^ phosphorylation does not affect p65 DNA binding (Fig. 7A and 7C) but has an important impact on gene transcription. Indeed we observe around two fold higher gene induction in cells expressing S547A mutated p65 compared to cells expressing wt p65. When we look at the basal gene expression level, an increased gene expression in cells containing S547A mutant p65 is also sometimes observed. This observation is not systematic and is probably due to a low basal level of stress in the cells. ATM is not only activated by DNA damage. Other stimuli such as hypotonic shock, oxidative stress and even TNFα treatment were shown to activate ATM [35], [36], [37]. It would be interesting to investigate whether ATM would also phosphorylate p65 following these stimuli and the subsequent impact of such a phosphorylation on gene transcription, compared to the present results with DNA damage. These investigations would also help in the understanding of the specificity of NF-κB response following different stimuli.

Comparison of sensitivity to TSA or promoter acetylation, of IL8 and IκBα, clearly show that the regulation mechanisms of these two genes are completely different (Fig. 8A–D). As expected given the results obtained from the microarray, IL8 expression regulation depends on Ser^547^ phosphorylation whereas for IκBα it does not. In addition to this regulation gene mechanism, IL8 and IκBα genes expression does not respond to TSA treatment in the same way in cells containing p65_wt_. Indeed, whereas TSA treatment has a no effect on IL8 expression, this treatment induces a significant decrease of IκBα mRNA level. This reveals an additional regulation mechanism for IκBα gene expression in response to etoposide. When we kook at the different studies on HDAC inhibitors, TSA could act at different levels to induce this decreased IκBα gene expression [38], [39], [40]. These observations indicate that probably several molecular regulation mechanisms coexist for the regulation of different NF-κB target genes, even in the same cell type and in response to the same stimulus.

In this study we highlight that Ser^547^ phosphorylation of p65 following etoposide enables interaction with HDAC1 which leads to histone deacetylation on gene promoter and subsequent decreased gene expression, but this phosphorylation could also play some other additional functions. It could displace a co-activator, such as HAT, recruitment or could be important for induction of other p65 post-translational modifications, such as acetylations. Indeed, some cases of crosstalk between phosphorylation and acetylation of p65 have been already described [16], [41]. p65 acetylations can either enhance or reduce NF-κB activity. Of note that, as phosphorylation, acetylation can modulate expression of only few specific target genes [16]. Ser547 phosphorylation of p65 could also interfere with p53 association. Indeed, it exist considerable crosstalk between these two transcription factors which can cooperate with each other for gene transcription under some circumstances [42], [43], [44]. The possible modulation of DNA damage-induced interaction between NF-κB and p53 by Ser^547^ phosphorylation of p65 on specific promoters could highlight an additional regulatory mechanism of gene expression. All of these last hypotheses would need further investigations to be elucidated.

Taking these results together, with the discovery of Ser^547^ phosphorylation of p65, we highlight a new, finer and specific, regulatory role of ATM in NF-κB regulation. It is now clear that p65 phosphorylations are important to determine its transcriptional functions on specific target genes through the modulation of interaction with co-activator or co-repressor. In this study, a decreased expression of a specific set of genes involved in inflammation due to recruitment of HDAC by phosphorylated p65 was observed. This is an additional regulatory mechanism controlling NF-κB response to DSB. Concerning clinical therapy based on the control of NF-κB activity, if we consider the possible use of drugs that selectively regulate NF-κB activity by affecting its post-translational modification instead of global NF-κB inhibitors, which have probably stronger side effects, this discovery would help in the development of such therapies.

## Materials and Methods

### Cell Lines and Culture and Reagents

Human embryonic kidney-293 cell line (HEK-293) (ATCC) and HEK-293FT (Invitrogen), were cultured in Dulbecco's Modified Eagle's medium (Lonza) supplemented with 10% fetal bovine serum (Lonza), 1% L-glutamine (Lonza), 1% NEAA (only for HEK-293FT) (Lonza), and 1% Penicillin/Streptavidine (P/S) (Gibco®, Invitrogen). Etoposide and TSA (Sigma) were used at a final concentration of 10 µM and 450 nM respectively, KU55933 (Merk) at a final concentration of 1 µM. Stock solutions of etoposide (10 mM), KU55933 (10,11 mM) were dissolved in DMSO and TSA (3,3 mM) in water.

### Antibodies

Anti-ATM (ab78) used for western blots was from ABCAM, anti-ATM (Ab-3, 819–844) used for ATM immunoprecipitation in the kinase assay from Calbiochem, anti-HDAC1 (sc-7872), anti-p65 (sc-109 or sc109x for supershift) and anti-p50 (sc-1191x) from Santa Cruz, anti-HA (HA.11 mAb) from Covance. Antibody used for ChIP assays were anti-histone H3 acetylated on Lys^9^ (Millipore 07–352), anti-p65 (Santa Cruz sc-109x) and anti-HDAC1 (Diagenode pAb-053-050).

### Plasmids and Site-directed Mutagenesis and Recombinant Proteins

Plasmid expressing GST-ATM_(1–247)_ was kindly provided by Professor Khanna KK [45]. Fragments of human p65 cDNA with the putative target Ser or Thr residue in its center were amplified by PCR and cloned in the pGEX-5X-3 (GE Healthcare), generating GST-p65_(105–157)_, GSTp65_(214–265)_, GSTp65_(348–400)_, GSTp65_(397–456)_, GSTp65_(521–551)_ fusion proteins. Duplex oligonucleotides corresponding to a fragment of p53 were hybridized and inserted in the pGEX-5X-3 to generate GSTp53_(9–22)_. HA-tagged human p65 was amplified by PCR from pCMV-HA p65 and cloned into pLenti6 Topo/D vector (Invitrogen). The reporter plasmid κB-Luc was from Stratagene. All mutants were generated by site-directed mutagenesis using the QuickChange Site-Directed Mutagenesis Kit (Stratagene) and verified by sequencing. ATM wt and kd purified recombinant proteins were kindly provided by Professor Paull T (Howard Hughes Medical Institute, Austin, Texas).

### Stable Cell Line

Lentiviral stocks were obtained by collecting supernatant of HEK-293FT cells, transiently co-transfected with the pLenti6 HA-p65 wt, S547A, or S547D, each containing silent mutations conferring resistance to an siRNA directed against endogenous p65, with the psPAX2 (Plasmid 12260, Addgene) and with the pVSV-G (a kind gift from Dr. T. Friedmann [46]). HEK-293 cells were next infected with the lentiviral stock and treated with Blasticidin (Invivogen) to select stably transduced HEK-293 cells expressing siRNA resistant HA-p65.

### Western Blotting and Electrophoretic Mobility Shift Assay (EMSA)

Cytoplasmic and nuclear extracts were prepared as previously described [47]. Cytoplasmic extracts were analyzed by Western blotting as described [48]. Nuclear extract were obtained and analyzed by EMSA as previously described [48] using ^32^P-labeled oligonucleotide κB or IL8 probes. The κB probe (5′-GGTTACAAGGGACTTTCCGCTG-3′; Eurogentec) corresponds to HIV-1 long terminal repeat κB site and the IL8 probe (5′-GGTTATCGTGGAATTTCCTCTG-3′; Eurogentec) corresponds to the NF-κB site of the IL8 promoter. Supershift experiments were performed as previously described [48].

### ELISA

The concentration of IL8 released in the culture media of treated and mock-treated cells was quantified using the Human IL8 ELISA Ready-SET-Go!® kit (eBioscience) according to the manufacturer’s recommendations.

### Yeast Two Hybrid Assay

pGBKT7 expressing GAL4 binding domain fused to ATM Heat repeat 1–2 (aa 1 to 150) was transformed into *Saccharomyces cerevisiae* HA109 strain. HeLa cDNA library, fused to the GAL4 activation domain into the pGADT7, was provided into *Saccharomyces cerevisiae* Y187 strain (BD Yeastmaker™, BD Clontech). After mating of the two yeast strains, co-transformants were plated on media lacking tryptophan, leucine and histidine, and, four days later, growing colonies at 30°C were screened with the X-Gal filter assay. Colonies transferred onto a filter were disrupted in liquid nitrogen and incubated with X-Gal 200 µL/mL in 30°C for several hours. Colonies that turned blue within 24 hours were scored as positive for interaction. The plasmids were purified from these selected colonies and the genes amplified by PCR and identified by sequencing.

### GST Pull-down Assay

Bacterially expressed GST or GST-ATM_(1–247)_ fusion protein were purified on glutathione agarose beads. Twenty four hour after transfection with pCMV-HA p65 wt, HEK-293 cells were lysed in LB buffer (50 mM Tris HCl, 150 mM NaCl, 2 mM EDTA, 1% NP-40, 10% glycerol, pH 8.00). One mg of protein was pre-cleared with beads-linked GST for 1 hour at 4°C. The pre-cleared lysate was next incubated with beads-linked GST-ATM or beads-linked GST alone as negative control for 2 hours at 4°C. The beads were then washed three times with LB buffer and the pulled down proteins were eluted with SDS loading buffer and subjected to SDS-PAGE.

### In vitro Kinase Assay

HEK-293 cells, were lysed in buffer (50 mM Tris HCl, 150 mM NaCl, 10% glycerol (v/v), 1% Tween-20, 1 mM DTT, 0,5 mM PMSF, 10 mM NaF, 50 mM β-glycerophosphate, 10 mM para-Nitrophenyl phosphate, Complete™ (Roche), pH 7,5). Aliquots of 750 µg of protein were pre-cleared with protein A-agarose (Pierce) for 1 h at 4°C. The pre-cleared lysates were next incubated in the lysis buffer with 1 µg of ATM antibody (Calbiochem) for 2 hours and for one additional hour with protein A-agarose at 4°C. The beads were then washed twice with the lysis buffer, once with LiCl buffer (100 mM Tris HCl, 0,5 M LiCl, pH 7,5), and twice with kinase assay buffer (10 mM HEPES, 50 mM NaCl, 10 mM MgCl_2_ and 10 mM MnCl_2,_ 1 mM DTT, 0,5 mM PMSF, 10 mM NaF, 50 mM β-glycerophosphate, 10 mM para-Nitrophenyl phosphate, Complete™, pH 7,5). Immunoprecipitated proteins were resuspended in 50 µl of kinase assay buffer, with or without ATM specific inhibitor KU55933 (1 µM), with 1 µl of [γ-^32^P]ATP (10µCi, 37.10^7^ mBq/mMol, Perkin Elmer) and with 1 µg of purified GST-p65 substrate. The mix was incubated 30 min at 30°C. The reaction was stopped by adding SDS loading buffer and was subjected to SDS-PAGE. Proteins were transferred onto PVDF membrane and autoradiographed.

### Co-immunoprecipitation

Cells were washed with cold PBS, scraped and centrifuged. Cells pellets were resuspended and incubated at 4°C for 30 minutes in IPLS buffer (20 mM Tris HCl, 120 mM NaCl, 0.5 mM EDTA, 0.5% NP40, 10% glycerol (v/v), 10 mM NaF, 50 mM β-glycerophosphate, Complete™, pH 7,5). Aliquots of equal amount of proteins were pre-cleared with protein G-agarose (Santa Cruz) for 1 h at 4°C.The pre-cleared lysates were next incubated in the IPLS buffer with 1 µg of HA antibody (Covance) overnight and for 2 additional hours with protein G-agarose at 4°C. The beads were then washed four times with IPLS buffer and the immunoprecipitated proteins were eluted with SDS loading buffer and subjected to SDS-PAGE.

### SiRNA Transfection

p65 siRNA (GCCCUAUCCCUUUACGUCA) [49], and control siRNA (*Silencer*® Select Negative Control #2 siRNA) were synthesized by Applied Biosystem and transfected into HEK-293 cells using ProFection Mammalian Transfection System – Calcium Phosphate (Promega). Cells were harvested 72 hours post-transfection. SiRNA efficiency was checked by western blotting.

### Luciferase Assay

Twenty four hours before treatment, HEK-293 cells were transfected with JetPEI (Lucron Bioproduct) with corresponding plasmid(s) (see Fig. 2) and two Luciferase reporter plasmids. The first one is under the control of a κB-dependent promoter and the second one (Renilla Luciferase) not. Eight hours after etoposide treatment, cells were lysed and assayed for luciferase activity with a Dual-Luciferase Reporter Assay System (Promega). The κB-dependent Luciferase activity was normalized with the Renilla Luciferase activity.

### Quantitative Real Time Reverse Transcription-PCR (qRT PCR)

Total RNA samples were extracted with RNeasy Mini Kit (Qiagen) according to the manufacturer’s recommendations. Reverse transcription and PCR were performed as previously described [38]. The primers used to analyze the different transcripts were designed with the software Primer Express™ (Applied Biosystems): *i*κ*b*α: FW (5′-CCAACCAGCCAGAAATTGCT-3′) and RV (5′-TCTCGGAGCTCAGGATCACA-3′); *IL8*, FW (5′-GAAGGAACCATCTCACTGTGTGTAA-3′) and RV (5′-ATCAG GAAGGCTGCCAAGAG-3′); SELE Fw (5′-TGTGAGGAAGGCTTCATGTTGC-3′) and Rv (5′-GCTGTGCACTGGAAAGCTTCA-3′); V-CAM Fw (5′-TATGTCAATGTTGCCC CCAG-3′) and Rv (5′-GATTTTCGGAGCAGGAAAGC-3′); CXCL1 Fw (5′CCACTGCGC CCAAACC3’) and Rv (5′TGCAGGATTGAGGCAAGCTT3’); A20 Fw (5′-TCTCGGCTAT GACAGCCATCA-3′) and Rv (5′-TCAAATCTTCCCCGGTCTCTG-3′); CD83 Fw (5′CCGCAGGTTCCCTACACGGT3’) and Rv (5′TGCTGTCCCCTGAGG TGGTCT3’); B2M Fw (5′-GAGTATGCCTGCCGTGTG-3′) and Rv (5′- AATCCAAATGCGGCATCT –3′) (Eurogentec).

### Microarray

Total RNA samples were extracted with RNeasy Mini Kit (Qiagen) according to the manufacturer’s recommendations. The yield of the extracted RNA was determined by measuring the optical density at 260 nm. The purity and quality of extracted RNA were evaluated using the Experion RNA StdSens Analysis kit (Bio-Rad Laboratories). High quality RNA with RNA Quality Indicator (RQI) score greater than 8 was used for microarray experiments. Gene expression profiling was performed using Illumina’s multi-sample format Human HT-12 BeadChip that contains more than 47000 probes and profiles twelve samples simultaneously on a single chip (Illumina). For each sample, 250 ng total RNA was labeled using an Illumina TotalPrep 96-RNA Amplification kit (Ambion) according to the manufacturer’s instructions. Briefly, double stranded cDNA was synthesized using T7-oligo (dT) primers and followed by an *in vitro* transcription reaction to amplify RNA while biotin was incorporated into the synthesized amplified RNA (aRNA) probe. The aRNA probe was then purified and quantified using a NanoDrop spectrophotometer (Thermo Fisher Scientific).

Biotinylated cRNA probe was hybridized to the Human HT-12 BeadChip Array (Illumina). Labeled aRNA (750 ng) was used for hybridization to each array. The hybridization, washing, and scanning were performed according to the manufacturer’s instructions.

The arrays were scanned using a BeadArray Reader (Illumina). The microarray images were registered and extracted automatically during the scan according to the manufacturer’s default settings. Raw microarray intensity data were analyzed with the GenomeStudio software normalized using the quantile normalization method according to the manufacturer’s recommendation. Differential analyses have been performed with Genome Studio software. The data have been normalized by quantile method and the differential analyses have been performed using the Illumina Custom model as differential expression algorithm. Significant differentially expressed probes are filtered with Diff score up to 30 or below -30 for up-regulated or down-regulated compared to reference, respectively. This threshold correspond to a p-value <0.001 for differential test. The biological relevance of up- and down-regulated genes were analyzed through the use of Ingenuity Pathways Analysis (Ingenuity® Systems, www.ingenuity.com).

### Chromatin Immunoprecipitation Assay (ChIP assay)

Chromatin immunoprecipitation (ChIP) assays were carried out as previously described [38]. The immunoprecipitated DNA was analyzed by quantitative real time PCR with the SYBR Green Master Mix in the ABI Sequence Detection System. The primers, corresponding to the promoter region of IL8 gene, were designed using the software Primer Express™^:^ FW (5′-GCCATCAGTTGCAAATCGTG-3′) and RV (5′-AGTGCTCCGGTGGCTTTT-3′) (Eurogentec).
